# Supporting professional development for educators during the pandemic: Lessons from an international blended learning diploma program

**DOI:** 10.1007/s11125-021-09591-5

**Published:** 2022-04-28

**Authors:** Enyi Jen, Sven Mathijssen, Lianne Hoogeveen

**Affiliations:** Radboud University Nijmegen (Radboud Universiteit), Nijmegen, Netherlands

**Keywords:** Professional development, Positive supervision, Gifted education, Blended learning, Educational technology

## Abstract

This article discusses issues that emerged from conducting professional development activities for educators during the Covid-19 pandemic. In 2020, as post-academic educational trainers, the authors worked closely with more than 200 professional educators who participated in an international diploma program to develop their professional skills and gain positive and insightful experience. Here, they share three lessons they learned while working with professional educators: (a) teachers face new challenges in the use of educational technology, (b) teachers wonder how to motivate learners with diverse abilities in a virtual setting, and (c) building a supportive community is essential. Suggestions and recommendations are also provided.

When Covid-19 changed the world dramatically in March 2020, almost every country enacted lockdown measures, including closing schools, to slow down the pandemic. Governments used executive orders to define who were essential workers, and teachers were defined as essential. When schools opened again in May 2020 (in the Netherlands), teachers were on the front lines. During the fall semester, the situation worsened, and some countries closed schools again. Teacher went back to online teaching. In some cases, even though instruction was primarily virtual, teachers had to go to a school building to take care of other essential workers’ children. In general, teachers experienced a challenging and stressful year, as pressure from their work added to the pressures of the pandemic.

UNESCO (2020c) emphasized that “teachers need access to relevant, quality professional development and support during crises: Teachers and education personnel need access to relevant, quality professional development and support to be able to continue teaching in crisis contexts” (p. 3). The concept that learning never stops is important not only for students but also for professional educators in kindergarten through grade 12 (UNESCO, 2020b).

 In 2020, as the post-academic educational trainers, we worked closely with more than 200 professional educators who participated in an international diploma program to develop their professional skills and gain positive and insightful experience. The Organization for Economic Co-operation and Development (OECD, 2020) stressed that both teachers and trainers need to remain up to date with the changing requirements of the modern workplace so they can address the current educational challenges adequately. Although the world is still in the Covid-19 pandemic, the field of professional development for educators should reflect on what has been learned and should use these lessons to strengthen future training. With this in mind, we share here the three main lessons we learned from working with educators in three international (i.e., Croatia, Malta, Nijmegen-international) and six domestic (e.g., the Netherlands) groups, and we provide reflections, accordingly.

## The new challenges of using educational technology

Before the Covid-19 pandemic, the use of blended learning (i.e., learning including both online learning and face-to-face instruction) in professional development has been discussed, and comments have usually been positive (e.g., Hew & Cheung, 2014; Jen & Hoogeveen, 2021). However, the current pandemic has exposed a new challenge associated with blending learning: screen fatigue. Online teaching and online learning require a significant amount of screen time and the huge amount of screen time consume much energy, which may cause the screen fatigue.

Our field experience offering various courses to professional educators suggests that three potential strategies can be adopted. First, educators can redesign some activities in their professional development plan. In our case, instead of hosting large-scale class discussions as we previously did, we divided the participants into groups and let them meet on their own schedule. This made their discussion easier. Second, educators can extend the study time of the course. For example, our seminar was held initially for 3 days. To extend that time, we opened an online learning environment for 2 weeks so participants could spread out their learning time. Third, educators can combine different online learning platforms. In the past, our participants were responsible for using a designated online learning platform (e.g., Brightspace, Blackboard). Now it is important for participants to be flexible with multiple platforms and online learning tools (e.g., Zoom, Microsoft Team).

## Teachers wonder how to motivate learners with diverse abilities in a virtual setting

The second lesson we learned is that participants were interested in discussing how to motivate and challenge their students in online learning, which was being used than before. Teachers also expressed concerns about how to keep high-ability students interested in learning. Studies have shown that although parents reported no difference in well-being during online teaching, compared with what they experienced during in-person classes, the motivation of their students declined during online teaching (Bakx et al., 2020a, 2020b). This topic is important because many high-ability programs were canceled during the pandemic. Whenever educators anticipate that their educational budget may be cut in the near future, they need to realize that the needs of high-ability learners may be overlooked.

UNESCO (2020a) created a comprehensive list with varied resources, applications, and online learning platforms as distance learning solutions. More specifically, the National Association for Gifted Children (NAGC, 2020) listed four tips to provide high-ability learners with remote instruction: enrich first, then accelerate; organization is key; communication is important; and use different tech tools with high-ability learners. Additionally, based on our teaching and research expertise, we suggest teachers help their high-ability students set up independent study projects, which provides these learners with the opportunity to develop and fulfill their talents. By doing independent studies, high-ability leaners can explore the topic they like in depth.

## The importance of building a supportive community

Professional development has been identified as a way to build a professional community. UNESCO (2020c) also proposed that professional development be used to transform teachers from crisis-affected communities into teacher-educators. Indeed, in our program, the participating professional educators supported each other in this community. They made connections between what they learned in the training and what they were experiencing in their current practices. They asked questions and exchanged useful tips, ideas, and resources.

Moreover, we adopted positive supervision (Bannink, 2015) in the training to help the participating professional educators focus on solution-building paradigms. Instead of only looking at and analyzing problems, the focus of the professional development was on looking at what worked and could be further built upon. Emotionally, in this community, we encouraged creativity, flexibility, and empathy. These so-called positive emotions and this type of support are exactly what educational communities need.

## Conclusion

The Covid-19 pandemic has made it even clearer that the use of blended learning is the future of education. We need to think carefully, however, about how to design blended training. Educators need to work in smaller subgroups, which give participants enough time to do their assignments, so they do not need to put in so many hours in front of their screen. In addition, a variation in learning platforms can prevent screen fatigue. Blended education is suitable for post-academic education, such as the professional training described in this article. However, teachers in primary and secondary education also need online learning tools to motivate and challenge their students, specifically high-ability students.

Both UNESCO (2020a) and NAGC provide teachers with information on how to meet the needs of students with diverse abilities in virtual settings. Independent study projects can be an alternative approach for high-ability learners to explore specific topics of interest. However, all decisions about what teachers provide should be based on their understanding of students’ abilities and interests. Thus, we stress the importance of helping teachers learn through professional development how to pre-assess their students’ abilities and interests effectively.

Supportive communities give teachers the possibility of encouraging each other. Our experience shows that this allows educators to make a connection between what they learn and what they experience in practice, so they can exchange tips, ideas, and resources. Positive supervision (Bannink, 2015) helps students and professionals focus on solution-building paradigms: what works and can be further built upon? Creativity, flexibility, and empathy are of paramount importance in education and should be encouraged throughout the educational process. We believe this kind of blended education will lead to the development of autonomous professionals in gifted education who support their students in fulfilling their potential and growing up as autonomous, happy adults.
